# An Essay on the Sensibility of Dentine and Its Treatment

**Published:** 1856-10

**Authors:** J. D. White


### ARTICLE IV.

*An Essay on the Sensibility of Dentine and its Treatment, read before the Pennsylvania Association of Dental Surgeons, December* 12, 1855. By J. D. White.

Mr. President and Gentlemen:—It has been a long time since I have had the honor of appearing before you in the capacity of an essayist, and with an eye ever directed to the investigation and discussion of practical subjects as best suited to our society gatherings, I have selected for the subject of a short essay, the Sensibility and Treatment of Dentine. The tubular structure of this substance renders it extremely difficult to manage its treatment for sensibility with such exactness as at all times to enable us to hold the treatment within such bounds as to obtain the most satisfactory results. Again, the conditions of the case, as to whether it is deep-seated or superficial decay, or in a younger or older tooth, or of a peculiar impressibility of the organ to the different substances employed for the purpose of treatment, it is necessary that great caution should be exercised, and a long experience enjoyed in practice, to insure invariable and successful results. It is doubtless out of these causes that so great a diversity of opinion grows as to the best course of treatment to pursue, or if any remedial means be employed or not. The different views advanced when this subject was under discussion before the late American Convention of Dentists, held in this city, fully establishes this fact. That there is an exquisite sensibility of dentine, daily experience shows; yet it is not universal, as some teeth can be cut with an instrument without exciting apparent suffering. And yet those teeth seem to possess a vital and healthy condition, and an internal pulp similar to those of a sensitive character. And again, there are different degrees of sensibility, varying from the bearable to the unbearable. This state of things does not admit of ready explanation, as it would seem to be rational that similar organs or tissues in health, would be endowed with analogous functions, and be impressed by similar stimulants. We do not seem to be capable, by practical tests, to push this peculiar condition of dentine to a different cause, although it seems fair to presume that it is invariably due to the irritation of nervous fibre. The same patient does not at all times suffer the same degree of pain of the teeth, by the friction of the instruments of the dentist, or by taking acid substances into the mouth; nor do all the teeth in the same mouth, or in the same state of decay, possess the same sensibility. This diversity of condition, and uncertainty of treatment, perplexes the impatient operator and causes him to abandon any attempt at treatment by medicaments, and imposes the whole burden of success consequent upon the treatment of those sensitive organs, to the endurance of the suffering patient. An ingenious theory, advanced by Dr. Goddard of our city is, that the sensibility of dentine is due to *concussion*, the fluids contained in the tubuli of the dentine being given off by, and resting upon the investing and sensitive membrane of the pulp, become disturbed by friction passing over their outer extremities, is transmitted to the surface of the pulp and induces pain. This theory would seem, by many cases, to gain some force, from the fact, that if we attempt to excavate a tooth when the cavity is very wet, it is more painful than when it is dry. The friction of the excavator tends to dry the cavity, and if we cease cutting when the operation is but half accomplished and the fluids of the mouth are permitted to pass in, it will be found to have taken an increased sensibility. Hence, a number of cases may be successfully treated by observing the precaution of keeping the cavity dry. This may possibly be explained, however, by the probability that the nerves in the tissue are in a more impressible or normal condition, while wet, than when dry. And again, the nerves when cut off when dry may contract and constantly keep retreating below the surface and keep out of the way of the instrument until the cavity is prepared. It is difficult to understand how every case that is met with can be explained, however, by either of the above named processes, or without admitting the actual presence of nervous tissue, but by being of such extreme delicacy as to be exceedingly transient and to be modified by such varying and undefinable conditions as to evade the scrutiny of the observer as to what the modified conditions are actually due. At one time of life this tenderness of the teeth may be extreme, and may disappear for a term of many years, and reappear as before. A case of this kind came under our notice about fourteen years ago. A gentleman of about forty years of age applied to us for advice, suffering from extreme sensibility of the necks of all his teeth; the cavities of decay were unusually tender, and contained a humid character of decay. The health of the patient was delicate from intense application to business. His former dentist had abandoned his teeth and informed him that he would inevitably lose them all. We treated a number of his teeth for this tenderness. The patient visited a southern climate for several winters for his health, which improved, and the tenderness left his teeth entirely, and they ceased decaying for a number of years, except forming a few cavities on the approximal surfaces, but which could always be operated on without pain. This condition lasted for about ten years, when the tenderness of the teeth returned, and with it a rapid process of decay. The old surfaces are softening, and the slightest exposure of new surfaces develops the extreme sensibility of their former condition. This case would favor the inference that the sensibility depended for its sensible development more upon the condition of the impressibility of the nervous system of the patient, than upon the presence or absence of nerves.

Escharotics, which act upon the animal tissues of other parts of the body, are more successful in treating this condition of dentine than sedatives. Opium or morphia may be applied for a long time without producing any sensible result, therefore *arsenious acid*, *caustic potash*, *lunar caustic*, *nitric acid* and *chloride of zinc*, as well as the *actual cautery* have in turn been freely employed. Among those substances arsenious acid is the most potent, reliable and painless where it can be employed with safety, such as in superficial cavities. But it is imprudent to apply it to a cavity that nearly reaches the pulp, or where it cannot be seen by the operator in from twelve to eighteen hours, as it will permeate any thickness of dentine if left on the part for a longer time, and produce inflammation of the nervous pulp. We prefer to apply it dry on cotton, or, if the cavity is very shallow and on the labial surfaces of the teeth, then we apply a portion of it in powder to the part, and spread over it a thin coating of white wax; or, where that cannot be done, we place over the part a lock of dry cotton-wool and throw a ligature around the tooth and secure it in that manner at least until the cavity can be excavated sufficiently to secure it with wax or cotton. It is not safe to apply the arsenic until the sensibility is totally gone, as it will be found after a time that the parts have absorbed too much, and the pulp will become inflamed a few days after the operation is considered to have been perfect. It frequently happens that when the protection to the arsenic has been removed, that the sensibility has not been much altered. In such a case let the cavity be exposed a while to the air and fluids of the mouth, and it will be found to have changed sufficiently to allow of easy excavation. Some times, when operating on the front teeth, we wait a few days, leaving the cavity open, in order to make sure that too much shall not be applied. It often happens, too, that after a partial suspension of sensibility has been effected by the arsenic that the chloride of zinc will finish the matter without exciting so much pain as if it had been employed at first. It is an error to apply arsenic combined with creosote, because the creosote is absorbed readily by the dentine, and without destroying the sensibility of the surface, especially if a small quantity be applied frequently, sufficient may be absorbed to inflame the pulp or irritate it and place the whole tooth in such a highly sensitive condition to heat and cold and to the touch, as to render it impossible for the tooth to be operated upon. If, under such circumstances, the tooth should redden in color, it should be drilled open and left without applying any thing until it had entirely recovered its normal sensibility; a neglect of this precaution even in treating the exposed pulp, when one application does not destroy its vitality, renders the use of the arsenic an objectionable substance in our operations. When excavating a cavity after applying arsenic, we do not feel safe until we cut down sufficiently to impinge upon a sensitive surface, for fear that we have left enough arsenic in the dentine below our plug to inflame the pulp when it approaches that substance. It might be said why employ a substance so dangerous in its tendency, and by which a tooth, by the slightest circumstance or uncertainty in using it, may be lost, or, at least, endanger the loss of its pulp? We would answer, that we believe that more teeth are lost, and more pulps exposed by defective plugging, consequent upon a too sensitive condition of the organ to be properly plugged, than by injudiciousness in the use of arsenic. We have been told this day by two patients that they never could keep plugs in their teeth until the nerves became exposed, on account of the fact that they could not bear the operation of properly cleansing a plugging, and we are now treating four front cavities that have been plugged frequently by a distinguished dentist, in which the plugs would not stay because the cavities could not be shaped to hold them, on account of too great a sensibility of the parts. Teeth are frequently found to be too sensitive between, to be filed or chewed upon, or be picked out or kept free from foreign substances; we treat these cases with arsenic to great advantage. Chloride of zinc, with us, takes the place of either of the other substances named. Where this will not act, neither of the other substances will. It will not blacken the teeth as the nitrate of silver, nor is it as irritable to the gums and mouth generally as the nitric acid or the caustic potash. In some teeth this substance acts like a charm and only by exciting a slight pungent or warm kind of pain. We give the history of some cases of this kind, where it proved invaluable, in the News Letter of October, 1854. Yet in some teeth it excites the most intense pain, without obtunding the tenderness of the parts in the slightest degree. We never employ the chloride of zinc for destroying the exposed nervous pulp, as has been inadvertently reported of us in the American Journal of Dental Science, July, 1855. But that we have used it for many years with advantage in the treatment of sensitive dentine is certain, and of which we have spoken elsewhere, and therefore think it needless to consider the subject any further at this time.—*News Letter.*

